# The effect of grassland type and proximity to the city center on urban soil and vegetation coverage

**DOI:** 10.1007/s10661-023-11210-z

**Published:** 2023-04-20

**Authors:** Hassanali Mollashahi, Magdalena Szymura, Peliyagodage Chathura Dineth Perera, Tomasz H. Szymura

**Affiliations:** 1 Institute of Agroecology and Plant Production, Wrocław University of Environmental and Life Sciences, Grunwaldzki Sq 24a, Norwida St. 25, 50-363 Wroclaw, Poland; 2 Department of Ecology, Biogeochemistry and Environmental Protection, University of Wrocław, Wrocław, Poland

**Keywords:** Ecotoxicity, Soil chemical properties, Urban grasslands, Ecosystem services

## Abstract

Urban soils with associated vegetation are important components of urban ecosystems, providing multiple regulating and supporting ecosystem services. This study aimed to analyze the differences in the soil chemistry and vegetation of urban grasslands considering urbanization gradient and urban grassland type (UGT). We hypothesized that the chemical properties of soil, such as metal content, as well as vegetation traits, differ according to grassland type (lawns, grasslands in parks, grasslands on river embankments, and roadsides) and the location of grassland patches (city center versus peripheries). Our samples included 94 UGT patches which each patch represented by four square sampling plots sized 1 m^2^. The results showed high differentiation of measured traits unrelated to UGT and location. The exception was K content, with a relatively high concentration in lawns, and some metals (Cd, Cu, Pb, Zn), with higher concentrations in the city center than in the peripheries. We found two grassland patches located in the city center where the concentrations of Pb, Zn, and Cu exceeded the level authorized by Polish standards. In the case of vegetation traits, the variability was not structured considering the UGT and location of the patches, except for bare soil cover, which was higher in lawns in the city center compared to embankments in the peripheries. We observed correlations between vegetation traits and soil chemical properties. The vascular plant species richness decreased when N, P, and C content, along with an increase in grass cover and a decrease in herbs.

**Supplementary Information:**

The online version contains supplementary material available at 10.1007/s10661-023-11210-z.

## Introduction

The urban ecosystem services provided by soil are associated with supporting roles (e.g., habitat for soil organisms) and a set of regulation services, such as nutrient and pollutant retention and release, carbon sequestration, and water storage (Calzolari et al., 2020), and regulation of the hydrologic cycle and infiltration of precipitation, where a lack of infiltration causes rapid formation of urban streams (Yeakley, 2014). Soil-based ecosystem services are associated with properties such as soil texture and nutrients (Adhikari & Hartemink, 2016). Additionally, the soil can filter pollutants from runoff waters, which carry chemical contaminants such as heavy metals, hydrocarbons, excess nutrients, pharmaceuticals, and personal-care products (Wessolek et al., 2011). Together with plants, soil contributes to the reduction of noise pollution (Derkzen et al., 2015). Urban soil and vegetation contribute to climate regulation by reducing the concentration of CO_2_ and other greenhouse gases in the atmosphere. Therefore, owing to ongoing climate change, more attention is being paid to sustainable soil management in urban areas. However, there is a lack of awareness of soil function from citizens and city planners (Lal & Stewart, 2017).

Many properties of urban soil fundamentally vary from their non-urban counterparts (Mónok et al., 2021). Urban soils are mostly technosols, while soils in urban gardens can be considered anthrosols because of long and intensive cultivation. In urban areas, building activities lower the soil quality, as they contaminate soils due to the debris from demolition. Building materials such as concrete and mortar contain calcium carbonate can make urban soils more alkaline than expected (Kida & Kawahigashi, 2015). Urban soils are usually compacted, which prevents carbon accumulation and is a cause of organic-matter depletion (Lal & Stewart, 2017). Compared with non-urban soils, the soil in urban areas is polluted and contains less organic matter. Soil organic pollutants weaken the mineralization process of plant litter (Vodyanitskii, 2015) and also the biochemical processes mediated by microorganisms (Falkowski et al, 2008). The concentrations of major nutritive elements such as N, P, and K are low in urban soils (Guilland et al., 2018). Deficiencies in soil organic carbon and nutrients (N, P, and K) can reduce soil microbial activity, contributing to poorer soil quality in urban areas. Due to its thin topsoil and less plant litter, urban soil has lower levels of organic matter, which reduces the amount of soil organic acids and elevates soil pH level (Day et al., 2010; Yeakley, 2020; Mónok et al., 2021).

The atmospheric wet-depositions of inorganic nitrogen (N), calcium (Ca^2+^), and magnesium (Mg^2+^) may have beneficial effects on plants and microorganisms (Lovett et al., 2000), while the long-term fertilization of urban lawns increases the soil nutrient content and humus amount (Ignatieva et al., 2020).

Urbanization and vehicle emissions increase the toxic compounds in soils (e.g., heavy metals), which pose a significant risk to human health (De Miguel et al., 2006; Vrščaj et al., 2008). Despite the widespread use of lead-free gasoline, lead (Pb) can still be emitted from engines and catalysts (Guan et al., 2017), industrial emission, and atmospheric deposition of coal combustion products (Nawrot et al., 2020), which reaches high levels near roads (Day et al., 2010). High metal concentrations (especially Pb, Cu, and Cd) adversely affect soil-living organisms especially the microbial parameters of soils (Papa et al., 2010; Ren et al., 2021). As a result, heavy metal concentrations are also used as an urban soil quality index (Mamehpour et al., 2021). Human activities, such as industrial activities, as well as factors such as atmospheric deposition and urban heat island effects (Lehmann & Stahr, 2007), increase nitrogen deposition, air pollution, water runoff, and soil pollution, and they reduce vegetation cover, which impacts soil-living organisms and biodiversity (Guilland et al., 2018).

Numerous ecosystem services provided by urban soils are tied to urban vegetation (Ignatieva et al., 2015; Derkzen et al., 2015; Lal & Stewart, 2017; Onandia et al., 2019; O’Riordan, 2021). In contemporary cities, the so-called urban grasslands are an important type of urban vegetation (Ignatieva et al., 2015). Traditional urban lawns are defined as patches of turf-type grasses that coalesce spatially into a distinct vegetation type (Thompson & Kao-Kniffin, 2017). Recently, due to the appreciation of the ecosystem services delivered by different forms of vegetation, urban grasslands are considered more broadly, encompassing meadows and lawns in domestic gardens, parks, vacant land, remnants of rural landscapes, and areas along transportation corridors (Onandia et al., 2019), including even non-grassy vegetation (Ignatieva & Hedblom, 2018; Smith et al., 2015). Many ecosystem services delivered by urban grassland are related to their biodiversity (Onandia et al., 2019; Thompson & Kao-Kniffin, 2017), and the structure of the vegetation is strongly shaped by human activities, such as fertilization level, mowing frequency, irrigation, trampling, and disturbance (Ignatieva et al., 2015; Ignatieva & Hedblom, 2018). As a result, the vegetation in a city center can differ from that in the peripheries (Deák et al., 2016; Vega & Küffer, 2021).

The worldwide trend of urbanization is increasing the urban land area; thus, it is imperative to understand the characteristics, changes over time, and management options of urban soil. For this purpose, soil characteristics, especially chemical properties (e.g. pH, nutrient/elemental imbalance, and soil C pools), must be duly considered for different types of plant-occupied soil (Fung et al., 2021; Lehmann & Stahr, 2007).

This study aimed to analyze the differences in the soil chemistry and vegetation traits of urban grasslands while considering urbanization gradient and grassland type. It could be assumed that the soil chemical properties, as well as the vegetation traits, differ among the UGT (lawns, parks, embankments, and roadsides) and the location of the patches (city center versus periphery, as reflecting urbanization gradient). Therefore, we hypothesized: (1) the patches in the city center are nutrient-poor but with a higher pH and are more contaminated by heavy metals compared to patches located in city peripheries, (2) the soil in lawns is more nutrient-rich due to fertilization compared to other urban grassland types, such as parks, road verges, and river embankments, and (3) road verges have a higher amount of heavy metals compared to other urban grassland types due to high traffic. We will also determine whether there is a correlation between vegetation traits and soil properties. To test our hypotheses, we analyzed the chemical properties of the samples, including the semi-total metal content of urban soils collected from different types of urban grasslands, and we also assessed vegetation characteristics, including the total vegetation cover, the coverage of different plant groups (grasses, herbs, mosses), bare soil cover, litter cover, and the number of vascular plant species in the studied plots.

## Material and methods

This survey was conducted in 2020 in the city of Wrocław, Lower Silesia, Poland (51° 6′ 28.3788′′ N, 17° 2′ 18.7368′′ E). The city’s population is approximately 650,000, and the total urban area is approximately 300 km^2^. The city is located at altitudes ranging from 105 to 156 m above sea level along the Odra River Valley. Because of the city’s location in the river valley, the city’s shape is rather elongated. The annual sum of precipitation is 548 mm, with most occurring as rainfall in the summer, and the average annual temperature is 9.7 °C, with July being the warmest month and January being the coldest (https://bip.um.wroc.pl/artykuly/196/o-wroclawiu). Urban grasslands, mostly in form of public and private lawns, road verges, and grasslands on river embankments, altogether cover 9523 ha, which constitutes 32% of the entire city area. The grassland patches are scattered all over the city, but they are more frequent in the northern part and the peripheries. The median area of the urban grassland patches is 0.4 ha. The smallest patches, with areas as high as 0.5 ha are the most numerous, particularly in the city center (Mollashahi et al., 2020). The grasslands in parks, roadsides, and some lawns are managed by the City Greenery Board; the grasslands along the watercourses are managed by the Regional Water Management Authority in Wrocław, while the remaining urban lawns are managed by numerous owners. The grasslands managed by public institutions are not fertilized, while the private grassland’s fertilization is dependent on the owner’s decisions. Also, sprinkling roads with salt in winter is common.

### Experimental design

**Fig. 1:**
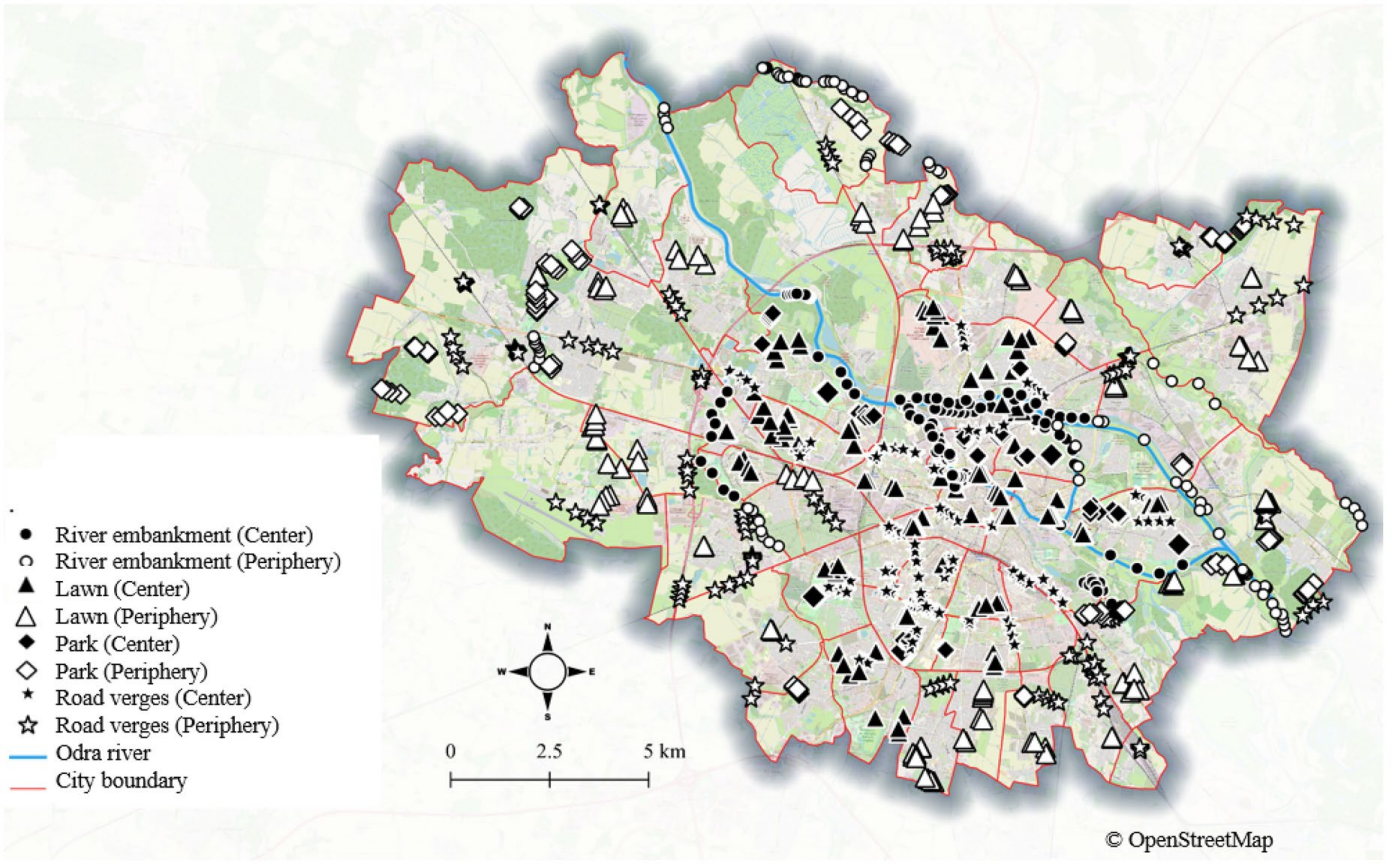
Distribution of sampling plots within Wroclaw City; each point represents 1 plot, which is equal to 1 m^2^. The background layer: OpenStreetMap contributors (https://www.openstreetmap.org/copyright)

The selection of patches for soil sampling was based on our previous investigation (Mollashahi et al., 2020). The different urban grassland types (UGTs), including road verges (R), embankments (E), parks (P), and lawns (L), were considered (Table 1). The patches were also classified according to their location: in the city center (C) and periphery (P). Because of the elongated city shape, classifying areas as the city center and periphery was based on both the geographical location of a particular district and information on population density. Districts with a population density above 2500 persons per ha were considered city center (see: SI Fig. 2). Generally, each of the eight groups of urban grasslands (4 UGT × 2 localities) was represented by 12 patches. However, in the case of parks in the city center (CP), only eight parks with lawns were found. Additionally, in the case of lawns in the city center (CL) and embankments in peripheries (PE), 13 patches were sampled. Altogether, 94 patches of urban grasslands were sampled. The locations of the patches tended to be spatially balanced, and the patches were uniformly distributed throughout different UGTs (Fig. 1). Each patch was represented by four plots, each sized at 1 m^2^, placed at regular distances. Soil samples (approximately 200 g) were taken from each plot (at a depth of 15 to 20 cm from the surface) and then mixed into one sample representing a particular patch. Artifacts such as plastic and glass were removed from the samples. Next, the samples were dried at room temperature and then crushed and sieved (Ø 0.5 mm) for subsequent analyses.

**Table 1:** Types of analyzed grassland patches

**The combination of different patches** CE, CL, CR, PE, PL, PP, PR, CP		
**Typology**	**Description**	**Number of patches**
Location	City center (C): the geographic core of the city and human population density larger than 2500 persons per ha	*n* = 45
	Periphery (P): city peripheries and human population density smaller than 2500 persons per ha	*n* = 49
Urban grassland types (UGT)	Road verges (R) Embankment (E) Parks (P) Lawns (L)	*n* = 24 *n* = 25 *n* = 20 *n* = 25

The cover of vegetation was assessed using a visual method in percentage scale (Mueller-Dombois & Ellenberg, 1974); this included the total vegetation cover, as well as the grass cover, herb cover, mosses cover, bare soil cover, and litter cover for each plot. The vascular plant species richness (N) that occurred in each plot was also calculated.

### Soil chemical analysis

Standard methods were used for soil chemical analysis: Kiejdhal’s method for total nitrogen content (N %), the Egner–Riehm spectrophotometric method for available P (mg*kg^−1^); flame photometry for available K (mg*kg^−1^) using ammonium acetate (C_2_H_7_NO_2_), elemental analysis for C (%), and spectrophotometry with titanium yellows for available Mg (mg*kg^−1^); The flame photometer method was used for the available form of Ca (mg*kg^−1^), soil pH in water, and KCl by potentiometric method (Sparks et al., 2020). The C: N and N:P ratios are also considered for the statistical analysis. For N:P calculation, N % was converted to mg*kg^−1^.

### Metal analysis

The total concentration of metals including Cd, Pb, Zn, Cu, Mn, Al, and Fe was measured after microwave digestion with aqua regia (HCl:HNO3 ratio 3:1). In short, 1 g of soil sample was digested with 10 mL of aqua regia using a microwave oven, in high-pressure PTFE beakers (Medyńska & Kabała, 2010; Microwave Plasma Atomic Emission Spectroscopy (MP-AES, 2022) ﻿Agilent Technologies﻿). Extracts were filtered with Munktell No. 2 filters, grade 0.84 g/cm^2^ (Ahlstrom Munksjö, Helsinki, Finland), and diluted with distilled water to 50 mL. Metal concentrations in obtained extracts were analyzed on Microwave Plasma-Atomic Emission Spectrometer MP-AES 4200 (Agilent Technologies, Santa Clara, CA, USA). Provided results are means from triplicate measurements, with the relative standard deviation automatically calculated by MP Software. (Medyńska-Juraszek et al., 2020; Pueyo et al. 2008). Quality of determination has been monitored using soil reference materials (NIST-1515, IAEA-V-10) with a certified total content of trace elements being analyzed. This method has been internationally standardized under European regulations and Environmental Protection Agency directions (Pillay, 2020; Soodan et al., 2014). The results were compared with the Polish standard for the accumulation of hazardous elements (Dziennik Ustaw, 2016), the values are shown in (SI Table 2).

**Table 2:** Kruskal–Wallis ANOVA results (chi^2^, *p* value) for differences in chemical element content for different locations, urban grassland types, and the interaction of these factors (* during N:P calculation N % was converted to mg*kg^−1^)

	**Location**		**Urban grassland-type**		**Interaction**	
	**H (chi**^**2**^**)**	***p***	**H (chi**^**2**^**)**	***p***	**H (chi**^**2**^**)**	***p***
N [%]	2.05	0.15	4.78	0.19	8.49	0.29
P [mg*kg^−1^]	0.04	0.85	7.35	0.06	10.77	0.15
K [mg*kg^−1^]	0.00	0.96	15.82	0.00	16.72	0.02
pH (KCl)	0.13	0.72	0.68	0.88	1.10	0.99
pH (H_2_O)	1.85	0.17	1.23	0.74	4.99	0.66
C [%]	0.43	0.51	2.26	0.52	6.82	0.45
Mg [mg*kg^−1^]	0.03	0.86	3.90	0.27	11.99	0.10
Ca [mg*kg^−1^]	3.33	0.07	0.63	0.89	4.88	0.67
C/N	0.00	0.99	0.15	0.98	3.24	0.86
N/P*	0.01	0.88	6.71	0.08	10.25	0.17

### Statistical analysis

The normality of the data distribution was checked using the Shapiro–Wilk test. Because the distribution often differs from normal, and different forms of data transformation to obtain normality of the distribution not always were successful, the Kruskal–Wallis ANOVA by ranks, with multiple comparisons of the median as the post hoc tests were applied to check the significant differences between groups in median values. The correlations between the analyzed traits were checked using Spearman rank correlations. To elucidate general patterns of analyzed traits, variability of Principal Component Analysis (PCA) was performed. Prior to the PCA, the data were normalized, and the lacking data values in Cd content were subtracted by the iterative imputation approach. The number of the analyzed axis was chosen based on the “brocek stick” approach, and the first analysis reveal the ordination was strongly biased by two samples, 35 and 40; thus, they were removed from the final PCA analysis. The analysis was performed using Statistica (version 13) and Past software, with a significance level of *p* < 0.05.

## Results

### Soil chemical properties

**Fig. 2:**
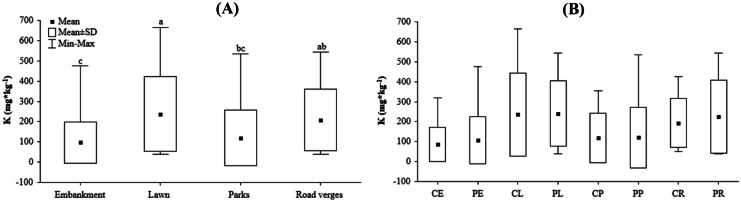
Differences in potassium content among soils from different urban grassland type (UGT) types (**A**) and the combination of localization and UG types (**B**). Abbreviations: city center (C), urban periphery (P), embankments (E), lawns (L), parks (P), and road verges (R)

The urban soil from grasslands in Wrocław had an almost neutral reaction (mean value pH_H2O_ = 6.93, pH_KCl_ = 6.37). The minimum soil pH _KCl_ and pH_H2O_ was approximately 5, and the maximum soil pH was from 7 to 8. There were no significant differences in the concentration of the analyzed elements or the ratios between them within grassland patches located in the city center or periphery, or belonging to different UGTs (embankments, parks, lawns, and road verges), except for the available K concentration (Table 2), which differed significantly among different grassland types and locations. The available K concentration was the highest in soils collected from lawns and the lowest in embankments soils, whereas road verges and park soils represented intermediate values (Fig. 2A). In the case of interaction, the post hoc tests were not able to detect differences between particular groups (Fig. 2B), contrary to the results of Table 2. Detailed data concerning the soil’s chemical properties are presented in (SI Table 1).

### Metal content in grassland soils

Generally, the soils from grasslands in the city center experienced higher metal deposition than those located in the urban periphery, especially grassland soil that occurred in road verges.

**Fig. 3:**
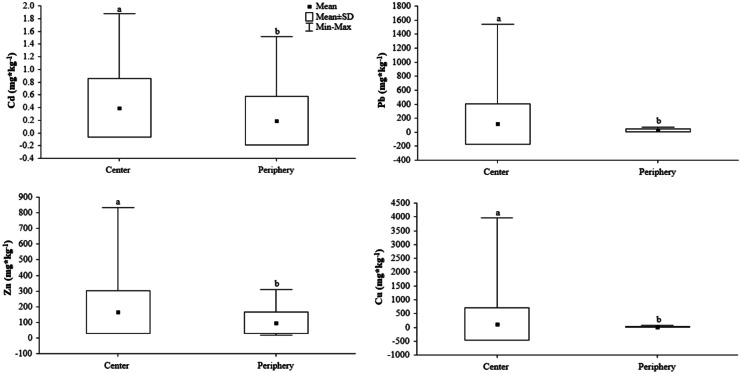
Differences in heavy metal (lead, cadmium, zinc, and copper) content in different sampling locations [city center (C) and periphery (P)] in the city

There were significant differences in the total concentration of metals, including Cd, Pb, Zn, and Cu (*p* value < 0.05), which depended on the patch location (center vs. periphery) and the interaction of location and UGT, whereas the total concentration of Cu and Mn varied with UGT (Table 3). The mean value of Cd, Pb, Zn, and Cu was higher in soil collected from plots located in the city center compared to that of the periphery (Fig. 3).

**Table 3:** Kruskal–Wallis ANOVA results (chi^2^, *p* value) for differences in heavy metal content for different locations, urban grassland types, and the interaction of these factors

**Variable**	**Location**		**Urban grassland-type**		**Interaction**	
	**H (chi**^**2**^**)**	***p***	**H (chi**^**2**^**)**	***p***	**H (chi2)**	***p***
Cd [mg*kg^−1^]	6.28	0.00	2.25	0.41	13.67	0.02
Pb [mg*kg^−1^]	25.22	0.00	5.88	0.12	32.03	0.00
Zn [mg*kg^−1^]	12.41	0.00	3.46	0.33	18.88	0.01
Cu [mg*kg^−1^]	12.65	0.00	10.01	0.02	25.25	0.00
Mn [mg*kg^−1^]	0.13	0.71	8.00	0.05	9.28	0.23
Al [mg*kg^−1^]	0.03	0.87	4.72	0.19	6.35	0.50
Fe [mg*kg^−1^]	0.78	0.38	3.87	0.28	5.64	0.58

**Fig. 4:**
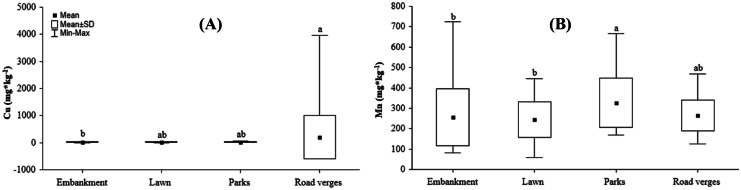
Differences in heavy metal content: **A** copper and **B** magnesium in different UGT

**Fig. 5:**
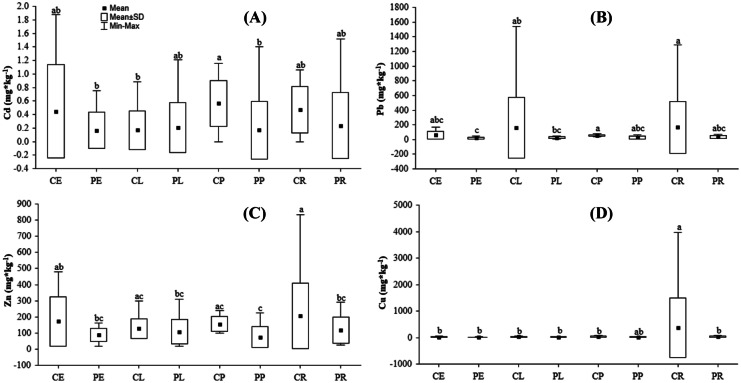
Differences in heavy metal content: **A** cadmium, **B** lead, **C** zinc, and **D** copper for the interaction of UGT and location of grasslands. Abbreviations: city center (C), urban periphery (P), embankments (E), lawns (L), parks (P), road verges (R)

The mean concentration of Cu in road verges was higher than that for other UGTs; however, it differed significantly from embankments (Fig. 4A). It was also observed that Cu concentration was higher in the soils of road verges in the city center than in other UGT located in the center and periphery; except for parks of periphery which shows the same mean value like road verges of the central part (Fig. 5D). The mean concentration of Mn was higher in park soils than in embankments and lawns (Fig. 4B). The mean concentration of Cd was higher in parks located in the city center than in parks and embankments located in the periphery and lawns in the central part of the city (Fig. 5A). The mean concentration of Pb was higher in road verges and parks located in the city center than in embankments and lawns in the periphery (Fig. 5B). The mean Zn concentration was higher in road verges in the city center and differed from other UGTs except for embankments and parks in the city center, and it was lowest in soils in parks located in the periphery (Fig. 5C). Basic descriptive statistics of metal content in the collected soils is presented in (SI Table 2).

### Vegetation coverage on urban grasslands

**Fig. 6:**
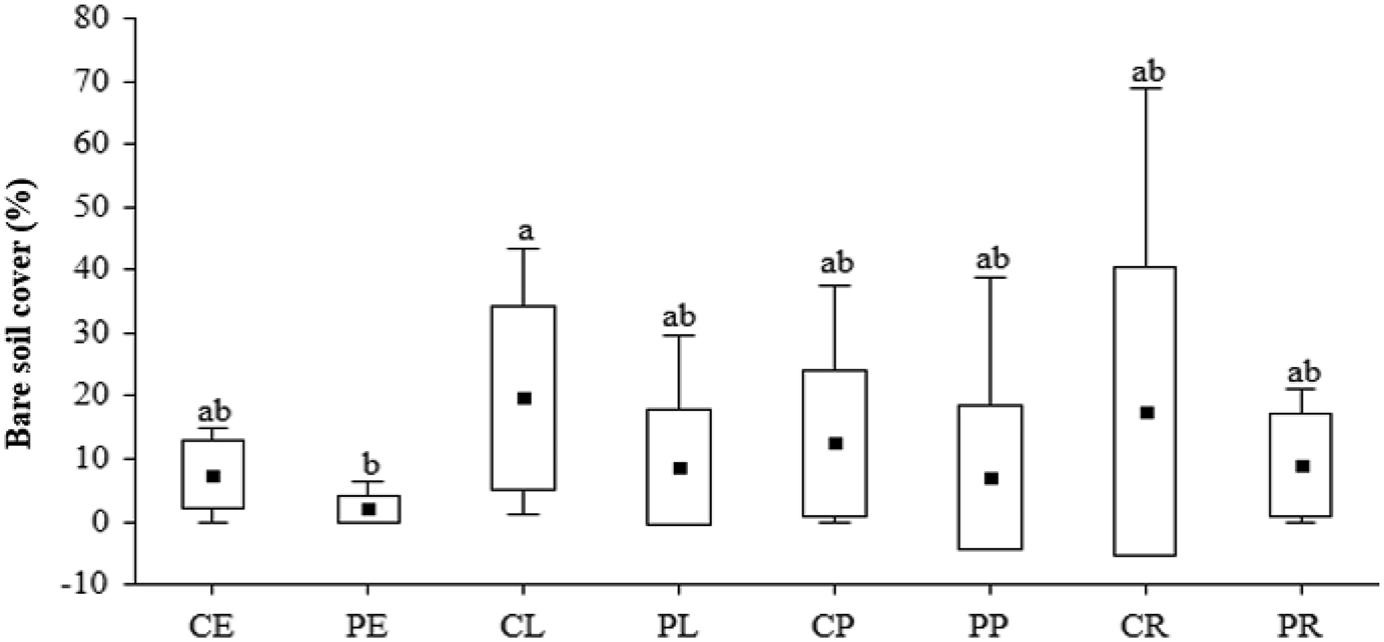
Differences in bare soil cover in grasslands located in the city center/periphery (**A**), different UGT (**B**), and interactions of location and UGT (**C**). Abbreviations: city center (C), urban periphery (P), embankments (E), lawns (L), parks (P), and road verges (R)

The average total vegetation cover for urban grassland was approximately 70% of the plots, bare soil cover was approximately 10%, and plant litter was approximately 20%; for details, see (SI Table 3). Within the average vegetation cover, the grasses had the highest average cover at 42%, followed by herbs and mosses at approximately 25% and 1.6%, respectively. The analysis indicated that the vegetation parameters did not differ among UGTs and locations, except for bare soil coverage, which was significantly higher in lawns placed in the city center (Table 4 and Fig. 6).

**Table 4:** Kruskal–Wallis ANOVA (chi^2^, *p* value) results for the vegetation coverage for different locations, urban grassland types, and the interaction of these factors

	**Location**		**Urban grassland-type**		**Interaction**	
	**H(chi**^**2**^**)**	***p***	**H (chi**^**2**^**)**	***p***	**H (chi**^**2**^**)**	***p***
Total vegetation cover (%)	0.71	0.40	7.25	0.06	8.61	0.28
Grass cover (%)	0.92	0.34	6.91	0.07	9.84	0.20
Herb cover (%)	0.16	0.69	4.47	0.21	9.93	0.19
Mosses cover (%)	0.42	0.44	1.95	0.40	2.90	0.74
Bare soil cover (%)	11.65	0.00	8.37	0.04	19.60	0.01
Litter cover (%)	1.49	0.22	1.40	0.71	5.01	0.66
Vascular plant species richness (N)	0.21	0.65	3.41	0.33	4.95	0.66

Among the observed traits (SI Fig. 1), we found a significant negative correlation of species richness with N, P, and C content in the soils, as well as grass cover, while there was a positive correlation with herb cover. Grass cover correlated positively with N content in the soils, while herb cover negatively correlated with N and K. We also observed significant positive correlations between herb cover and the content of heavy metals such as Cd, Pb, Zn, and Cu. The content of Cd, Zn, Mn, and Fe also correlated positively with the percentage of bare soil (SI Fig. 1).

**Fig. 7:**
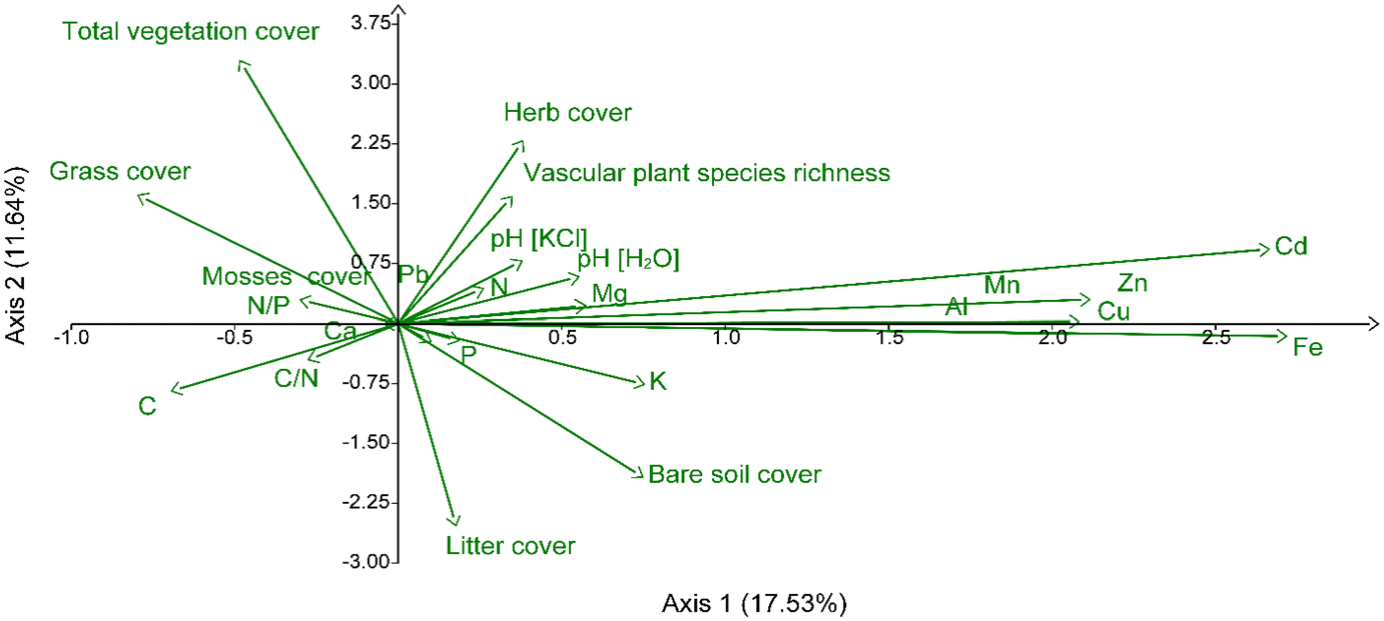
The biplot of two first axis of Principal Component Analysis

The first four axes of PCA explain 46.87% of the entire set data variation. The first PCA was correlated positively with the metal concentrations, except Pb (Table 5, Fig. 7). The second with the vegetation traits: the bare soil and litter cover was correlated negatively with species richness and plant cover. The third axis can be interpreted as an effect of N and K over fertilization, which increases grass cover while decreasing species richness and herbs and mosses cover. The N and K concentrations correlated also negatively with pH. The fourth axis reveals the effect of P concentration in soils, which influences the N/P ratio. This axis correlate also positively with pH but negatively with Ca concentration (Table 5, Fig. 7).

**Table 5:** The loadings values of particular variables in the Principal Component Analysis axis

**Variable name**	**PC 1**	**PC 2**	**PC 3**	**PC 4**
pH [KCl]	0.0640	0.1323	** − 0.2314**	**0.2890**
pH [H_2_O]	0.0933	0.0990	** − 0.2722**	**0.3878**
Cd	**0.4484**	0.1568	0.0274	** − **0.0065
Pb	0.0441	0.0741	0.0533	0.0383
Zn	**0.3562**	0.0505	0.1007	** − **0.0472
Cu	**0.3450**	** − **0.0222	0.0877	** − **0.0677
Mn	**0.3505**	0.0044	** − **0.0642	** − **0.0558
Al	**0.3346**	0.0003	0.0710	** − **0.1032
Fe	**0.4574**	** − **0.0261	0.0603	** − **0.0658
N	0.0004	** − **0.0052	**0.4724**	** − **0.0198
P	0.0314	** − **0.0357	0.1174	**0.5784**
K	0.1270	** − **0.1278	**0.2104**	0.1415
C	** − **0.1171	** − **0.1419	**0.2323**	** − **0.0558
Mg	0.0969	0.0375	0.1314	0.1944
Ca	0.0171	** − **0.0376	0.0857	** − 0.2101**
C/N	** − **0.0467	** − **0.0762	** − **0.1304	0.0319
N/P	** − **0.0074	** − **0.0009	** − **0.0634	** − 0.4739**
Total vegetation cover	** − **0.0816	**0.5553**	0.1516	** − **0.0149
Grass cover	** − **0.1343	**0.2710**	**0.4011**	** − **0.0123
Herb cover	0.0643	**0.3862**	** − 0.2222**	0.0391
Mosses cover	** − **0.0507	0.0501	** − 0.3752**	** − **0.1393
Bare soil cover	0.1262	** − 0.3238**	** − **0.1166	0.0959
Plant litter cover	0.0297	** − 0.4268**	** − **0.0996	** − **0.0622
Vascular plant species richness	0.0588	**0.2684**	** − 0.2449**	** − **0.1993
Explained variation [%]	17.53	11.64	10.04	7.66

The largest values (above absolute value 0.2) in a particular axis are highlighted in bold

## Discussion

Our results did not confirm our hypothesis regarding differences in macroelements and pH between particular UGTs and patch localities: the variability within distinguished groups exceeded the differentiation between UGTs and localities. The exception was available K concentration, but its variable pattern is not easy to explain. Intuitively, the higher K content in lawns can be related to their fertilization (Cekstere & Osvalde, 2013; Ignatieva et al., 2020), but in Poland, the typically used fertilizers are NPK (Gospodarczyk & Rutkowska, 2006). However, here, the observed patterns of P and N content are not correlated with K (SI Fig. 1); thus, it is unlikely that the high K levels result from lawn fertilization.

The range of pH variability in urban areas can be related to the effect of human activities. Mostly, the soils of Wrocław exhibited a nearly neutral pH, whereas according to majorities of studies, urban soil pH is slight to very strongly alkaline, which is typical of urban soils (Yang & Zhang, 2015). A neutral or moderately alkaline pH is beneficial to ecosystems since it can avoid the movement of hazardous elements such as heavy metals, which are dangerous to humans and beneficial organisms. At the soil-particle surface, trace metals are immobilized due to the high pH (Ge et al., 2000), where metal solubility decreases by increasing soil pH (Chuan et al., 1996). This also occurs because of a high cation exchange capacity that can bind contaminants (Kargar et al., 2015). A few plots exhibited an alkaline soil reaction, which can be attributed to the presence of construction materials in the soils, such as concrete and cement, contamination by ash from coal-fired powder, or even sands that are used for gritting (Birke et al., 2011; Gaberšek & Gosar, 2018). Rather, the presence of patches with acidic soils can be attributed to possible soil transportation from other, non-urban sites. Nonetheless, recent studies revealed that in urban regions, nitrogen (N) and sulfur (S) deposition could cause soil acidification, but these observations came from regions with a warm and humid climate (En-Qing et al., 2015; Huang et al., 2015), and they are rather unlikely to occur in a temperate climate. However, building materials such as concrete and mortar contain calcium carbonate, which can make urban soils more alkaline than expected (Asabere et al., 2018). The alkaline soil pH can negatively impact the plant and soil organisms in the cities, creating a habitat more suitable for non-native species (Delgado-Baquerizo et al., 2021; McKinney, 2006).

Contrary to macroelements and pH, we observed significant differences in metal concentration among the UGTs and locations of urban grasslands. The grassland soils in the central part of the city had a higher metal content, which can be attributed to urban activities. Human activities such as vehicular traffic, urbanization, and increased population density usually correlate with an increase in metal content in urban soil (Mónok et al., 2021; Argyraki & Kelepertzis, 2014). This was clear for road verges located in the city center. We linked this phenomenon to higher car traffic, which is an important source of metals (Adamiec et al., 2016; Napier et al., 2008; Silva et al., 2021). Typically, the main source of metals is brake and tire wear, motor oil, and traffic for Zn, brake wear, stop points, and traffic for Cu, tire wear for Cd, and brake, oil and additives, gasoline, and traffic for Pb, where high volumes of traffic occur (Bari & Kindzierski, 2017; Crosby et al., 2014; Ferreira et al., 2016; Hsu et al., 2017; Nawrot et al., 2020). Despite the use of unleaded gasoline, gasoline emission still was the main source of Pb in a study by Hong et al. (2018). In two patches belonging to road verges and lawns located in the city center, the concentration of Pb, Zn, and Cu even exceeded the background level authorized by the Polish standard for the accumulation of hazardous elements (SI Table 2, Fig. 2). This may increase the risk of human-related diseases in the long term (Lamas et al., 2016).

In the case of the vegetation, our observations suggest that the higher soil fertility, as expressed by nitrogen, phosphorus, and carbon amount, decreased species richness by increasing the cover of grasses at the expanse of herbs—such a phenomenon was reported for semi-natural grasslands (Ceulemans et al., 2013; Harpole & Tilman, 2007). The results suggest that physical disturbances of the vegetation coincide with contamination with metals, but they were beneficial for herbs. We did not have detailed data regarding plant species composition, but it can be assumed that strongly competitive grass species dominated the vegetation on more fertile and undisturbed sites, while disturbances causing the presence of bare soil which create empty niches for ruderal herb species (Nabe-Nielsen et al., 2021), which increase species richness in urban grasslands. Nonetheless, the observed regularities were rather independent of UGT and periphery, except for bare soil cover, which showed a higher percentage of lawns placed in the city center compared to embankments in peripheries and can be directly related to anthropogenic disturbances such as trampling by pedestrians (Wang et al., 2018).

## Conclusions

Obtained results show differentiation of soil and vegetation traits of urban grasslands, which is usually not structured regarding urban grassland type and patch locations (city center vs periphery). Considering the fertility of the soils, lawn soils contain only a higher concentration of K, than other UGT, but the value did not differ from road verges. A higher amount of heavy metals was detected in road verges located in the city center compared to other UGTs. The prominent, and rather easy-to-explain, patterns were exhibited by heavy metals whose concentrations were higher in the city center. The general correlations between vegetation traits and soil properties were mainly related to the decrease of biodiversity on UG with more fertile soils and the increase of herb and bare soil cover on UG with higher metal content. Moreover, the results suggest a positive effect of contemporary management on species richness, which allows establishing a herb species while increasing soil fertility to increase the cover of grass species leading to a decrease in total vascular plant species richness.

The observed concentration of heavy metals exceeded the allowed standards in patches located in the city center, suggesting the necessity of continuous monitoring of heavy metals in urban soils.

## Supplementary Information

Below is the link to the electronic supplementary material.

Supplementary file1 (DOCX 275 KB)

Supplementary file2 (DOCX 27 KB)

## Data Availability

All relevant data are within the manuscript and its Supporting Information files.
