# Supplementary material for: The effect of grassland type and proximity to the city center on urban soil and vegetation coverage
Source: Environ Monit Assess. 2023 Apr 20;195(5):599. doi: 10.1007/s10661-023-11210-z (PMC10119043; doi:10.1007/s10661-023-11210-z)
Supplement: Supplementary file 1 — Supplementary file1 (DOCX 275 KB) [file 10661_2023_11210_MOESM1_ESM.docx]

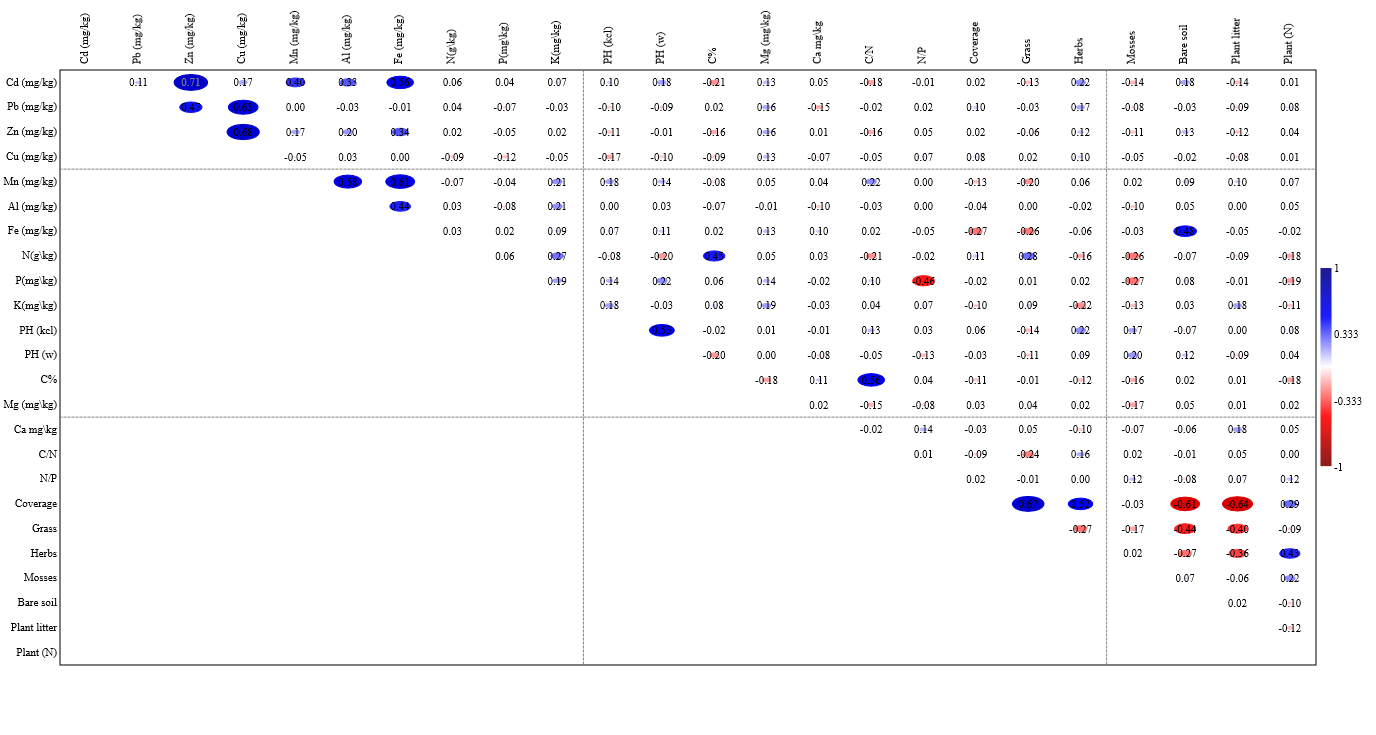


**Figure 1** Correlation matrix between total vegetation cover, soil chemical properties, and heavy metals. Plant (N): represents the number of vascular plant species richness.


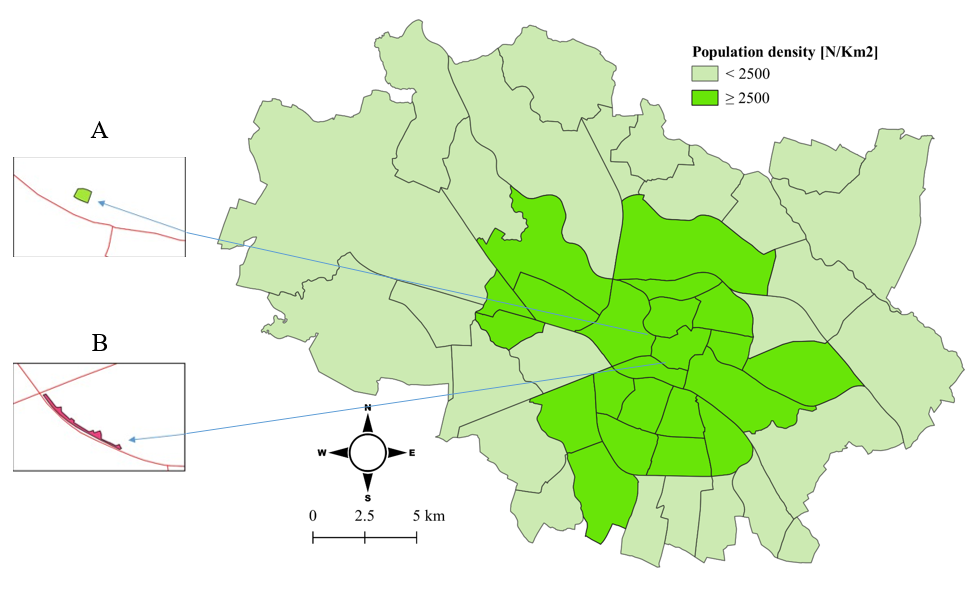


**Figure 2** District with population density above 2500 person per Km^2^ was considered as centre. Sites of grassland patches A: CR and B: CL, where the heavy metals including Pb, Zn, and Cu were higher than the Polish standard level; see Table 2 in supplementary material. CR refers to patches belongs to Road verges located in the City Center, and CL refers to patches belongs lawn located to the City Center.
