# Supplementary material for: The effect of grassland type and proximity to the city center on urban soil and vegetation coverage
Source: Environ Monit Assess. 2023 Apr 20;195(5):599. doi: 10.1007/s10661-023-11210-z (PMC10119043; doi:10.1007/s10661-023-11210-z)
Supplement: Supplementary file 2 — Supplementary file2 (DOCX 27 KB) [file 10661_2023_11210_MOESM2_ESM.docx]

**TABLE 1** Basic descriptive statistics of soil chemical properties of urban grassland patches.

| **Variable** | **Mean** | **Median** | **Minimum** | **Maximum** | **Std.Dev.** |
| --- | --- | --- | --- | --- | --- |
|  |  |  |  |  |  |
| N [%] | 1.39 | 1.30 | 0.29 | 2.46 | 0.51 |
| P [mg*kg^-1^] | 74.76 | 61.75 | 1.00 | 252.60 | 62.33 |
| K [mg*kg^-1^] | 167.82 | 80.00 | 20.00 | 665.00 | 157.38 |
| pH (KCl) | 6.37 | 6.44 | 5.00 | 8.20 | 0.64 |
| pH (H_2_O) | 6.93 | 6.97 | 5.05 | 8.95 | 0.64 |
| C [%] | 3.47 | 2.98 | 0.05 | 11.85 | 2.14 |
| Mg [mg*kg^-1^] | 98.31 | 102.07 | 16.75 | 164.19 | 33.18 |
| Ca [mg*kg^-1^] | 1852.6 | 1638.0 | 36.0 | 7160.0 | 1387.8 |
| C/N | 2.05 | 1.92 | 1.82 | 2.56 | 0.35 |
| N/P* | 94.39 | 23 | 1.9 | 1200 | 199.36 |

***** during N:P calculation N % was converted to mg*kg^-1^

**TABLE 2** Basic descriptive statistic of soil heavy metals of urban grasslands patches and standard level authorized by the Polish government (mg*kg^-1^).

| **Variable** | **Mean** | **Median** | **Minimum** | **Maximum** | **Std.Dev.** | Polish standard level**  (mg/kg) |
| --- | --- | --- | --- | --- | --- | --- |
| Cd [mg*kg^-1^] | 0.3 | 0.0 | 0.0 | 1.9 | 0.4 | 2 |
| Pb [mg*kg^-1^] | 70.3 | 36.0 | 3.0 | 1541.1 | 202.7 | 200 |
| Zn [mg*kg^-1^] | 129.7 | 105.5 | 17.9 | 834.0 | 110.7 | 500 |
| Cu [mg*kg^-1^] | 67.5 | 20.5 | 2.7 | 3963.5 | 406.8 | 200 |
| Mn [mg*kg^-1^] | 270.3 | 246.2 | 57.1 | 724.0 | 111.3 | *** |
| Al [mg*kg^-1^] | 7084.7 | 7072.5 | 1858.5 | 13450.0 | 2321.0 | *** |
| Fe [mg*kg^-1^] | 10504.8 | 9365.0 | 1892.0 | 53050.0 | 5786.8 | *** |

**From group ɪ, which is related to urban areas.

***These metals usually occur in soil in large values; they become toxic to plants and soil biota under low soil pH values.

| **Grouping variable** | **Mean** | **Median** | **Minimum** | **Maximum** | **Std.Dev.** |
| --- | --- | --- | --- | --- | --- |
|  |  |  |  |  |  |
| Total vegetation cover (%) | 69.4 | 75 | 0 | 97.5 | 21.2 |
| Grass cover (%) | 42 | 41.3 | 0 | 87.5 | 19.1 |
| Herb cover (%) | 26 | 22.5 | 0 | 67.5 | 15.8 |
| Mosses cover (%) | 1.6 | 0 | 0 | 20 | 3.5 |
| Bare soil cover (%) | 10.2 | 6.3 | 0 | 68.8 | 13 |
| Litter cover (%) | 19.1 | 15 | 0 | 73.8 | 14.8 |
| Vascular plant species richness (N) | 6.2 | 6 | 0 | 14 | 2.5 |

**TABLE 3** Basic descriptive statistics of vegetation traits of urban grasslands.
